# Injured Proximal Tubular Epithelial Cells Lose Hepatocyte Nuclear Factor 4α Expression Crucial for Brush Border Formation and Transport

**DOI:** 10.1016/j.ajpath.2025.01.011

**Published:** 2025-02-13

**Authors:** Michelle Kha, Ylva Magnusson, Iva Johansson, Gülay Altiparmak, Jaana Lundgren, Jenny Nyström, Kerstin Ebefors, Karl Swärd, Martin E. Johansson

**Affiliations:** ∗ Department of Laboratory Medicine, Institute of Biomedicine, Sahlgrenska Center for Cancer Research, Sahlgrenska Academy, University of Gothenburg, Gothenburg, Sweden; † Department of Clinical Pathology, Sahlgrenska University Hospital, Gothenburg, Sweden; ‡ Department of Physiology, Institute of Neuroscience and Physiology, Sahlgrenska Academy, University of Gothenburg, Gothenburg, Sweden; § Department of Experimental Medical Science, Lund University, Lund, Sweden

## Abstract

Recent studies have demonstrated that the transcription factor hepatocyte nuclear factor 4α (HNF4A) drives epithelial differentiation in the renal proximal tubules (PTs) and is critical for maintaining a mature PT phenotype. Furthermore, HNF4A down-regulation has been observed following kidney injury in mouse models. The aim of the present work was to investigate the role of HNF4A during acute and chronic human kidney disease and the loss of the mature PT phenotype in cultured PT cells. Loss of HNF4A expression and gain of vimentin expression were reciprocal and gradual during both acute and chronic kidney disease, as indicated by immunohistochemistry. Healthy human kidneys demonstrated partial or total loss of HNF4A expression in vimentin-positive scattered tubular cells. Primary cell isolation and subculture of PT cells recapitulated HNF4A-associated loss of the PT phenotype. Re-expression of HNF4A in cultured PT cells by adenoviral transduction increased transcripts related to brush border formation as well as absorptive and transport processes, as shown by RNA sequencing and gene set enrichment analyses. Thus, the reduction of HNF4A and increase of vimentin expression were connected to both acute and chronic kidney disease and represented a stereotypic injury response of the PT, resulting in dedifferentiation. HNF4A re-expression in cultured primary PT cells could provide a more reliable and predictive *in vitro* model to study PT function and injury.

## Graphical abstract

**Figure:**
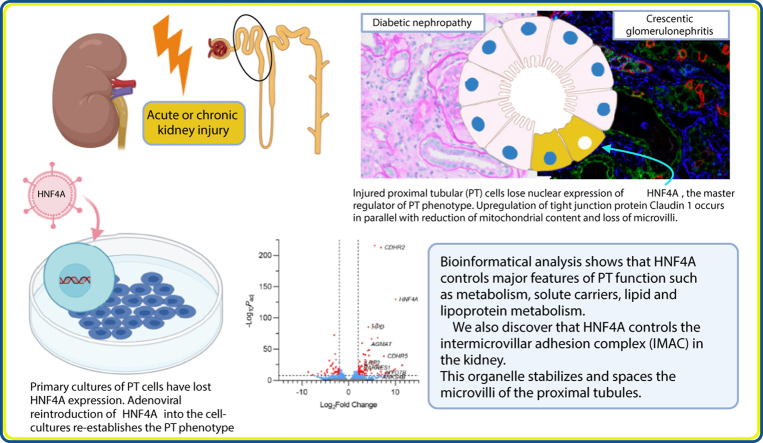

The primary glomerular and tubulointerstitial diseases add to major global diseases, such as diabetes and hypertension, to cause kidney disorder at an immense scale. Furthermore, acute kidney injury occurs in >20% of hospitalized patients.^1^ This results in a staggering number of patients experiencing chronic kidney disease, estimated to affect approximately 10% of the global population.^2^ Regardless of cause, it is often the resultant impact on the tubular compartment that determines the ultimate effect on kidney function. One reason is that the proximal tubules (PTs) are dependent on a continuous nutrient and oxygen supply because of their high metabolic activity. This renders the PT cells particularly sensitive to ischemic or toxic injury. Normally quiescent, tubular cells display a robust capacity for regeneration following injury, by dedifferentiation followed by proliferation.^3^ However, the mechanisms connecting injury and regeneration are still incompletely understood. A cell population termed scattered tubular cells (STCs), associated with kidney injury and regeneration, is found in a scattered pattern throughout the PT of the human kidney.^4^, ^5^, ^6^, ^7^ Whether the STCs represent a progenitor cell population or an induced phenotype triggered by injury is debatable.^4^^,^^5^^,^^8^, ^9^, ^10^, ^11^, ^12^, ^13^ The STCs express markers such as vimentin (VIM) and SRY box transcription factor 9 (SOX9), and are characterized by a dedifferentiated phenotype, lacking brush border and mitochondria.^6^^,^^14^^,^^15^ Recent studies show that they may represent a heterogeneous population in a transient state, with different subsets being involved in repair as well as stress and fibrogenic pathways.^15^, ^16^, ^17^ One of the subpopulations, referred to as failed repair or maladaptive proximal tubule cells, characterized by expression of vascular cell adhesion molecule 1 (VCAM1), is associated with a proinflammatory cell state.^13^^,^^16^^,^^18^^,^^19^ Further investigation is needed to identify how these subpopulations are induced and interrelated, and how they contribute to repair of the PT.

The transcription factor hepatocyte nuclear factor 4α (HNF4A) is a key regulator of PT development, controlling the expression of genes central for PT function, such as transport and metabolism.^20^ Furthermore, HNF4A is required for brush border formation, a structure essential for the reabsorptive function of the PT cells.^20^^,^^21^ Reduced HNF4A activity is observed following ischemia-reperfusion injury in mouse models,^18^^,^^19^ in parallel with a general down-regulation of normal PT gene expression.^13^^,^^22^ Single-cell sequencing of human kidney shows that VCAM1-positive cells, so-called failed repair proximal tubule cells, have reduced expression of HNF4A.^16^ Furthermore, PT cells lose their typical expression pattern during culture, including key genes involved in transport and metabolism.^23^, ^24^, ^25^ Instead, the cells start to express injury markers associated with the STC phenotype.^15^ The present study was designed to examine the relationship between loss of HNF4A expression and concurrent development of the STC phenotype, represented by induction of VIM expression. This transition of phenotype was studied in biopsies of human kidney disease and in cultured primary PT cells. Moreover, whether the PT phenotype could be restored in cultured primary kidney cells by forced HNF4A overexpression followed by transcriptomic analyses was also investigated.

## Materials and Methods

### Tissue Procurement and Cell Culture

Human kidney tissue was procured following written informed consent from the patients and with permission from the Swedish Ethical Review Authority (GU413-09, LU680-08, and 2020 to 06242). Sections from diagnostic kidney biopsies from workup of human kidney diseases were obtained, and clinical data were collected from the patient records. Diseases were crescentic glomerulonephritis (*n* = 6), IgA nephropathy (*n* = 6), and diabetic nephropathy (*n* = 6). A summary of the clinical data is presented in Table 1. For the biopsy study, normal tissue sections were aquired from pre-implant biopsies (*n* = 6). Normal kidney tissue for cell culture experiments was obtained from kidneys that were surgically removed because of kidney cancer at Sahlgrenska University Hospital (Gothenburg, Sweden). Following surgery, the kidneys were transported on ice for examination by an experienced pathologist (M.E.J.). Material for experimental purposes was collected from the kidney tissue localized farthest from the tumor.

**Table 1:** Summary of Patient Data

Patient no.	Diagnosis	Age, years	Sex	Serum albumin, g/L	Plasma creatinine, mmol/L	Plasma urea, mmol/L	Blood pressure, mmHg	Urinary albumin/creatinine ratio	Urinary albumin, mg/mmol creatinine
1	CN	42	M	25	330	12.8	143/88	79	531
2	CN	68	F	31	132	19.1	135/80	23	89
3	CN	68	M	29	170	14.8	136/79	334	3275
4	CN	73	M	37	392	32.3	195/93	84	1048
5	CN	68	F	20	330	18.4	145/85	612	5661
6	CN	79	F	31	276	17.2	160/73	44	189
7	IgAN	73	F	17	118	9.4	140/75	947	7388
8	IgAN	19	F	26	68	4.6	137/87	79	1304
9	IgAN	30	M	33	131	7.6	145/85	245	2261
10	IgAN	56	M	40	109	6.6	132/75	32	245
11	IgAN	21	M	39	88	5.2	143/79	59	1307
12	IgAN	66	M	37	212	25.8	148/91	86	458
13	DN	33	M	20	67	4.8	150/90	485	989
14	DN	61	M	27	193	9,7	210/110	345	2638
15	DN	71	F	39	167	16,3	136/59	344	3318
16	DN	53	M	30	195	12,3	135/80	399	1208
17	DN	51	M	34	182	14	158/94	210	1111
18	DN	28	M	14	204	13	150/92	1120	4164

F, female; M, male; CN, crescentic nephritis; DN, diabetic nephropathy; IgAN, IgA nephropathy.

Primary proximal tubular epithelial cells (PTECs) were obtained as previously described.^15^ In brief, kidney cortex tissue was cut into smaller pieces and incubated at 37°C with 2 mg/mL collagenase type I (C0130; Sigma-Aldrich, St. Louis, MO) and 40 U/mL DNase (D4263; Sigma-Aldrich) in Dulbecco’s modified Eagle’s medium low-glucose medium (31885023; Gibco, Waltham, MA) during agitation. The cells were further treated with red blood cell lysis buffer, followed by 0.125% trypsin. The cell suspension was filtered through 40- and 20-μm cell strainers and cultured in Dulbecco’s modified Eagle’s medium low-glucose medium supplemented with 10% fetal bovine serum (10091148; Gibco) and 1% penicillin-streptomycin (15140122; Gibco) in an atmosphere of 5% CO_2_ and 95% humidity at 37°C.

Human renal proximal tubular epithelial cells (CC-2553; Lonza, Basel, Switzerland) were purchased and cultured in REGM Renal Epithelial Cell Growth Medium BulletKit (CC-3190; Lonza), according to the manufacturer's instructions, in an atmosphere of 5% CO_2_ and 95% humidity at 37°C.

### Histology and Immunohistochemistry

Kidney tissue was fixed in formalin and embedded in paraffin according to standard procedures. Primary PTECs and Lonza renal PTECs were fixed in formalin and embedded in paraffin using the Cellient Automated Cell Block System (Hologic, Marlborough, MA). Tissue and cell blocks were sectioned at 4-μm thickness. Deparaffinization, rehydration, and antigen retrieval were then performed using EnVision FLEX Target Retrieval Solution (high pH) in a Dako PT Link instrument (Agilent, Santa Clara, CA). Histopathologic evaluation was performed on sections stained with hematoxylin and eosin or periodic acid–Schiff staining. Immunohistochemistry was performed using EnVision FLEX in a Dako Autostainer (Agilent), following the manufacturer's protocol. For immunohistochemical double staining, the same protocol was used with diaminobenzidine as the chromogen, followed by 0.3 mol/L sulfuric acid (GC203; Agilent) wash. Next, the protocol was repeated for the second primary antibody with magenta as the chromogen. The following primary antibodies were used: HNF4A (1:500; 3113; Cell Signaling Technology, Danvers, MA), VIM (1:1000; ab45939; Abcam, Cambridge, UK), and CD10 (ready-to-use; IR648; Agilent).

### Correlative Light and Electron Microscopy

Kidney tissue was fixed in 4% formaldehyde and 0.5% glutaraldehyde in 0.05 mol/L cacodylate buffer. The tissue was washed in 0.05 mol/L cacodylate buffer and incubated with 50 mmol/L glycine. Dehydration and embedding in LR Gold Resin (AGR1284; Agar Scientific, Stansted, UK) was performed using the progressive lowering of temperature technique in Leica EM AFS (Automatic Freeze Substitution System; Leica Microsystems, Wetzlar, Germany). In the final steps of resin infiltration, 0.5% benzoin ethyl ether was added as a UV initiator, followed by UV polymerization. The tissue was sectioned at 500-nm thickness on gelatin-chromium-carbon–coated slides. Immunohistochemistry was performed as described using VIM (1:50; ab45939; Abcam) as primary antibody. Light microscopy images were acquired using ZEISS Axio Imager. Z2 (Carl Zeiss, Oberkochen, Germany) and ZEN 2.6 (blue edition) software (Carl Zeiss). Slides were contrasted with 4% osmium tetroxide, 2% uranyl acetate, and Reynold's lead citrate stain, followed by scanning electron microscopy image acquisition using ZEISS Gemini II 450 (Carl Zeiss) and Atlas 5.0 software (Carl Zeiss).

### Colocalization Studies of Mitochondria, Actin-Associated Proteins, and Intercellular Junctions

Tissue from renal biopsies diagnosed with crescentic glomerulonephritis or diabetic nephropathy had been fixed in neutral-buffered formalin and embedded in paraffin, according to standard protocol. Blocks were sectioned at 3-μm thickness. Antigen retrieval was performed by boiling for 10 minutes in 10 mmol/L sodium citrate buffer, pH 6, followed by an equilibration period of 20 minutes at room temperature. Tissues were blocked by 30-minute incubation with Protein Block (catalog number X0909; Agilent). The following antibodies were used: goat anti-VIM (1:200; AF2105; R&D Systems, Minneapolis, MI); rabbit anti–claudin 1 (CLDN1; 1:200; ab211737; Abcam), mouse anti-mitochondrial marker (1:200; ab92824; Abcam); mouse anti–filamin A (FLNA; 1:200; AHO1402; ThermoFisher Scientific, Waltham, MA); and mouse anti-zyxin (ZYX; 1:300; MA5-31367; ThermoFisher Scientific). Primary antibodies were diluted in 4% normal donkey serum in phosphate-buffered saline and allowed to incubate for 60 minutes. Secondary antibodies were donkey anti-goat 647 (A21447), donkey anti-rabbit 594 (A21207), and donkey anti-mouse 488 (A21202), all from Invitrogen (Waltham, MA). Secondary antibodies were diluted in phosphate-buffered saline and allowed to incubate for 60 minutes. Mounting medium containing DAPI was added before coverslipping (Prolong Gold with DAPI; P36934; ThermoFisher Scientific). A confocal microscope, ZEISS LSM800, was used to capture images with using the ZEN 2.6 software, blue edition (Carl Zeiss).

### Adenoviral Transduction

Primary PTECs were seeded at a cell density of 125,000 cells per well in 6-well plates 1 day before transduction. PTECs were transduced at 10 multiplicity of infection with Human HNF4A Adenovirus (AD07632Z; Creative Biogene, Shirley, NY) or Null Adenovirus (AD00303Z; Creative Biogene) as control in Dulbecco’s modified Eagle’s medium low-glucose medium supplemented with 10% fetal bovine serum and 1% penicillin-streptomycin. Cells were harvested after 4 days for RNA and 8 days for protein. For long-term experiments (>4 days), cells were retransduced every third to fourth day.

### RNA Extraction, Reverse Transcription, and Quantitative PCR

RNA was extracted as previously described.^15^ Reverse transcription was performed using the RevertAid RT Reverse Transcription Kit (K1691; ThermoFisher Scientific), following the manufacturer's instructions. Quantitative PCR was performed using SYBR Green PCR Master Mix (4309155; Applied Biosystems, Waltham, MA) on an Applied Biosystems 7500 Fast Real-Time PCR System. Expression levels for *AGMAT*, *ANKS4B*, *CDHR2*, *CDHR5*, *HNF4A*, *HPD*, *LRP2*, *MYO7B*, and *RARRES1* were normalized using two housekeeping genes, *HMBS* and *RPL13A*, and determined using the 2^−ΔΔC_T_^ method (where C_T_ is threshold cycle). The primer sequences are shown in Table 2.

**Table 2:** Sequences of Primers for Quantitative PCR

Genes	Forward primers	Reverse primers
*AGMAT*	5′-GTATGATCTTTCTGGGAACAC-3′	5′-TTTGTCTTGAAGAGCACAAG-3′
*ANKS4B*	5′-GATAATCTGCAGTAGAGGAGG-3′	5′-TCTGTAAGTCATTATCCAGGG-3′
*CDHR2*	5′-AGTGTCATCATAGGATTGGG-3′	5′-GCTCAGTGTTGTACATGTTAG-3′
*CDHR5*	5′-ATTACGAGGAGAAGTCACTG-3′	5′-ATCTCCTTGGTCTTAAAGGG-3′
*HNF4A*	5′-AGTACATCCCAGCTTTCTG-3′	5′-AATGTAGTCATTGCCTAGGAG-3′
*HMBS*	5′-GGCAATGCGGCTGCAA-3′	5′-GGGTACCCACGCGAATCAC-3′
*HPD*	5′-AATGGTACCTGAAAAACCTG-3′	5′-CCGTTATAGTCCACATATTCC-3′
*LRP2*	5′-GCCGATGCATTTATCAAAAC-3′	5′-TCACATCCATCTTCATCTCC-3′
*MYO7B*	5′-CATACACTCAGAAGCAAGTC-3′	5′-CAGTTAACAGCCAAGATCAG-3′
*RARRES1*	5′-CAAGAATCAGAAACCCAGAC-3′	5′-TCAGGTATGCTGACTATTTCC-3′
*RPL13A*	5′-CCTGGAGGAGAAGAGGAAAGAGA-3′	5′-TTGAGGACCTCTGTGTATTTGTCAA-3′

*AGMAT*, agmatinase; *ANKS4B*, ankyrin repeat and sterile α motif domain-containing 4B; *CDHR2*, cadherin-related family member 2; *CDHR5*, cadherin-related family member 5; *HNF4A*, hepatocyte nuclear factor 4α; *HMBS*, hydroxymethylbilane synthase; *HPD*, 4-hydroxyphenylpyruvate dioxygenase; *LRP2*, low density lipoprotein receptor-related protein 2; *MYO7B*, myosin VIIB; *RARRES1*, retinoic acid receptor responder 1; *RPL13A*, ribosomal protein L13a.

### Western Blot Analysis

Protein lysates were obtained using radioimmunoprecipitation assay lysis buffer (20 to 188; Millipore, Burlington, MA) with the addition of Protease Inhibitor Cocktail Set III (539,134; Millipore), and protein concentration was measured using the Pierce BCA Protein Assay Kit (23,227; ThermoFisher Scientific). Protein lysates were loaded at 30 μg in NuPAGE 4% to 12% Bis-Tris Mini Protein Gels (NP0322BOX; Invitrogen) using MOPS SDS Running Buffer (NP0001; Invitrogen) or NuPAGE 3% to 8% Tris-Acetate Mini Protein Gels (EA03752BOX; Invitrogen) using NuPAGE Tris-Acetate SDS Running Buffer (LA0041; Invitrogen). The iBlot Transfer Stack (IB401002; Invitrogen) was used for transfer on the iBlot Gel Transfer Device (Invitrogen). The polyvinylidene difluoride membrane was washed with tris-buffered saline with Tween 20 (SC-281695; Santa Cruz Biotechnology, Dallas, TX) and blocked with 5% milk in tris-buffered saline with Tween 20, followed by incubation with primary antibodies overnight at 4°C. The following primary antibodies were used: agmatinase (AGMAT; 1:1000; ab231894; Abcam), ankyrin repeat and sterile α motif domain-containing 4B (ANKS4B; 1:1000; PA5-48711; ThermoFisher), cadherin-related family member 2 (CDHR2; 1:1000; NBP1-92246; Novus Biologicals, Centennial, CO), HNF4A (1:1000; 3113; Cell Signaling Technology, Danvers, MA), 4-hydroxyphenylpyruvate dioxygenase (HPD; 1:1000; ab133515; Abcam), low-density lipoprotein receptor-related protein 2 (LRP2; 1:1000; ab76969; Abcam), and retinoic acid receptor responder 1 (RARRES1; 1:1000; ab154742; Abcam). After washing in tris-buffered saline with Tween 20, the membranes were incubated with Goat anti-Rabbit IgG (H + L) Secondary Antibody, HRP (1:10,000; 31,460; ThermoFisher Scientific) or Goat anti-Mouse IgG (H + L) Secondary Antibody, HRP (1:1000; 32,430; ThermoFisher Scientific) for 2 hours at room temperature. Bands were visualized using SuperSignal West Dura Extended Duration Substrate (37,071; ThermoFisher Scientific) and developed using the Amersham ImageQuant 800 system (Cytiva, Chicago, IL).

### RNA Sequencing and GSEA

After HNF4A transduction, RNA from four biological replicates was sequenced using Illumina (San Diego, CA) NovaSeq 6000 at the Genomics Core Facility at Sahlgrenska Academy, University of Gothenburg, resulting in 60.7 million to 66.6 million 2 × 100 bp reads. The quality of the data was assessed using FastQC version 0.11.2 (Babraham Bioinformatics, *https://www.bioinformatics.babraham.ac.uk/projects/fastqc*). Quality trimming of reads was performed using Trim Galore version 0.4.0 (*https://github.com/FelixKrueger/TrimGalore*) and Cutadapt version 1.9.^26^ Data were mapped to the human reference genome from the Ensembl database (GRCh38) using STAR version 2.5.2b (*https://github.com/alexdobin/STAR?tab=readme-ov-file*). Mapped reads for each gene were counted using featureCounts version 1.6.4.^27^ Differentially expressed gene (DEG) analysis was performed using DESeq2 version 1.38.3^28^ in R version 4.2.2 (*https://www.r-project.org*). Adjusted *P* < 0.05 was considered statistically significant.

Gene Set Enrichment Analysis (GSEA)^29^ was performed using R version 4.2.2 and the R package clusterProfiler version 4.4.4.^30^ Genes in the list of DEGs with adjusted *P* < 0.001 and |log_2_ fold change| > 2 were considered significantly overrepresented. GSEA was performed using Gene Ontology (GO) terms within the biological process and cellular component domains and illustrated with dot plots. In addition, GSEA was performed using GOrilla (*https://cbl-gorilla.cs.technion.ac.il*),^31^ where genes in the list of DEGs were ranked based on log_2_ fold change and adjusted *P* value and separated by up-regulation and down-regulation with a rank cutoff of 5. For enrichment plots, a ranked list of DEGs based on log_2_ fold change and adjusted *P* value was used as input. The analyzed gene sets were GO terms from GSEA or gene sets supplied by the Molecular Signatures Database (*https://www.gsea-msigdb.org/gsea/msigdb*,^32^, ^33^, ^34^ last accessed May 28, 2024). Gene sets were preranked using gseapy prerank and plotted using gseaplot in Python version 3.10.11.^35^ False discovery rate (FDR) < 0.25 was considered statistically significant.

### Statistical Analysis

Data are presented as means ± SEM. Statistical analysis was performed using paired *t*-test in GraphPad Prism 9 (GraphPad Software, San Diego, CA). *P* < 0.05 was considered statistically significant (∗*P* < 0.05, ∗∗*P* < 0.01, ∗∗∗*P* < 0.001, and ∗∗∗∗*P* < 0.0001).

## Results

### Proximal Tubular Cells Display Extensive Loss of HNF4A Expression during Kidney Disease

**Figure 1:**
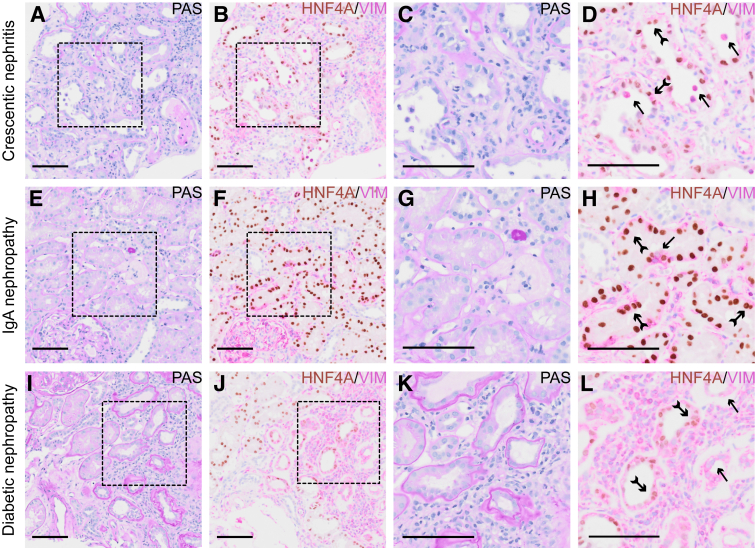
Injured proximal tubules lose hepatocyte nuclear factor 4α (HNF4A) expression during kidney disease. **A:** Periodic acid–Schiff (PAS) staining of human kidney biopsy from patient experiencing crescentic nephritis. **B:** Immunohistochemical double staining of crescentic nephritis for HNF4A (brown) and vimentin (VIM; pink). **C** and **D:** Magnified images of **A** and **B**, respectively, display HNF4A^−^/VIM^+^ cell detachment into the lumen (**arrows**) and HNF4A^+^/VIM^+^ cells in the proximal tubules (**fletched arrows**). **E:** PAS staining of human kidney biopsy of IgA nephropathy. **F:** Immunohistochemical double staining of IgA nephropathy for HNF4A and VIM. **G** and **H:** Magnified images of **E** and **F**, respectively, demonstrate expanded VIM expression through the injured proximal tubules, where some cells are lacking HNF4A expression (**arrow**), although most cells are double positive (**fletched arrows**). **I:** PAS staining of human kidney biopsy from a patient with diabetic nephropathy. **J:** Immunohistochemical double staining of diabetic nephropathy for HNF4A and VIM. **K** and **L:** Magnified images of **I** and **J**, respectively, reveal complete loss of HNF4A in atrophic flattened tubules (**arrows**), whereas weak HNF4A expression is retained in less injured tubules (**fletched arrows**). VIM expression is widespread. Quantification of HNF4A^+^/VIM^−^, HNF4A^+^/VIM^+^, and HNF4A^−^/VIM^+^ proximal tubular cells and number of replicates are presented in Table 3. Scale bar = 100 μm (**A**–**L**).

HNF4A is a transcription factor that governs epithelial differentiation of the PTs. In normal kidney, most PT cells display positive nuclear staining for HNF4A, as shown in immunostainings from the Human Protein Atlas version 23 (*https://www.proteinatlas.org/ENSG00000101076-HNF4A/tissue/kidney*,^36^ last accessed May 28, 2024). To investigate the status of HNF4A during disease, histologic material was examined from patients with crescentic nephritis (representing severe acute kidney injury), diabetic nephropathy (as an example of chronic kidney disease), and IgA nephropathy (representing phenotypically variable acute or chronic disease at moderate intensity). Consecutive sections of kidney biopsies were stained with periodic acid–Schiff and immunohistochemical double staining for HNF4A and VIM, an established kidney injury marker. Quantification of HNF4A^+^/VIM^−^, HNF4A^+^/VIM^+^, and HNF4A^−^/VIM^+^ PT cells was performed by manual counting (Table 3). In cases affected by crescentic nephritis, most glomerular corpuscles displayed active cellular crescents (Figure 1A). Moreover, the PTs displayed nonisometric vacuolization and sloughing of the apical membranes as signs of acute cellular injury. VIM expression was induced in approximately 50% of the PT cells (Figure 1B). Expression of HNF4A was retained in a proportion of the VIM-positive cells, albeit with considerable variability. Approximately 10% of PT cells were HNF4A^−^/VIM^+^, and were frequently found in the process of basal membrane detachment and sloughing into the lumen (Figure 1, C and D), whereas HNF4A^+^/VIM^+^ cells were attached to the tubular basal membranes. Biopsies from patients with IgA nephropathy were chosen to represent extended but low-level injury compared with crescentic nephritis. All cases were characterized by a generalized mesangial expansion and occasional synechiae of the capillary loops of the Bowman capsule (Figure 1E). The tubules displayed cytoplasmic vacuolization and slight reduction of epithelial cell volume and expanded VIM expression (Figure 1F). Approximately 15% of the PT cells co-expressed HNF4A and VIM (Figure 1, G and H), but near 10% of the PT cells displayed VIM positivity and complete loss of HNF4A. Finally, the cases of diabetic nephropathy were characterized by mesangial expansion, nodular sclerosis, and arteriolar hyalinosis (Figure 1I). The degree of tubulointerstitial fibrosis ranged between 30% and 50%, and in the more severely injured tubular segments, stretches of flattened cells were observed in atrophic tubules. VIM expression was widespread in the flattened tubules, whereas HNF4A expression was weak or most often lost (Figure 1J). A magnified view of the atrophic tubules showed gradual HNF4A reduction (Figure 1, K and L) and some tubules with complete loss of HNF4A. The number of HNF4A^+^/VIM^+^ PT cells was near 20%, whereas HNF4A^−^/VIM^+^ PT cells accounted for approximately 15% of PT cells. Taken together, these results demonstrate that HNF4A expression is gradually reduced during kidney disease progression, with a parallel gain in VIM expression. Severe acute and chronic kidney injury results in similar and surprisingly widespread responses, but with the important observation that loss of HNF4A^−^/VIM^+^ PT cells into the lumen is associated chiefly with acute injury.

**Table 3:** Quantification of Proximal Tubular Cells from Immunohistochemical Double Staining for HNF4A and VIM

Kidney disease	Patient	HNF4A^+^/VIM^−^, %	HNF4A^+^/VIM^+^, %	HNF4A^−^/VIM^+^, %
CN	CN1	55	23	22
	CN2	52	42	7
	CN3	49	46	5
	CN4	57	37	6
	CN5	60	32	8
	CN6	76	20	4
IgAN	IgAN1	88	9	2.6
	IgAN2	84	13	2.8
	IgAN3	62	23	15
	IgAN4	87	7	6
	IgAN5	72	25	3
	IgAN6	81	15	4
DN	DN1	52	31	17
	DN2	71	12	18
	DN3	76	14	11
	DN4	80	17	3
	DN5	73	22	5
	DN6	82	18	2
N	N1	94	5.3	0.7
	N2	92	3.4	4.6
	N3	98	1.8	0.25
	N4	93.6	6.0	0.44
	N5	96	3	1
	N6	95	4	1

CN, crescentic nephritis; DN, diabetic nephropathy; HNF4A, hepatocyte nuclear factor 4α; IgAN, IgA nephropathy; N, normal kidney; VIM, vimentin.

### Scattered Tubular Cells of Normal Human Kidney Display Reduced HNF4A Expression

**Figure 2:**
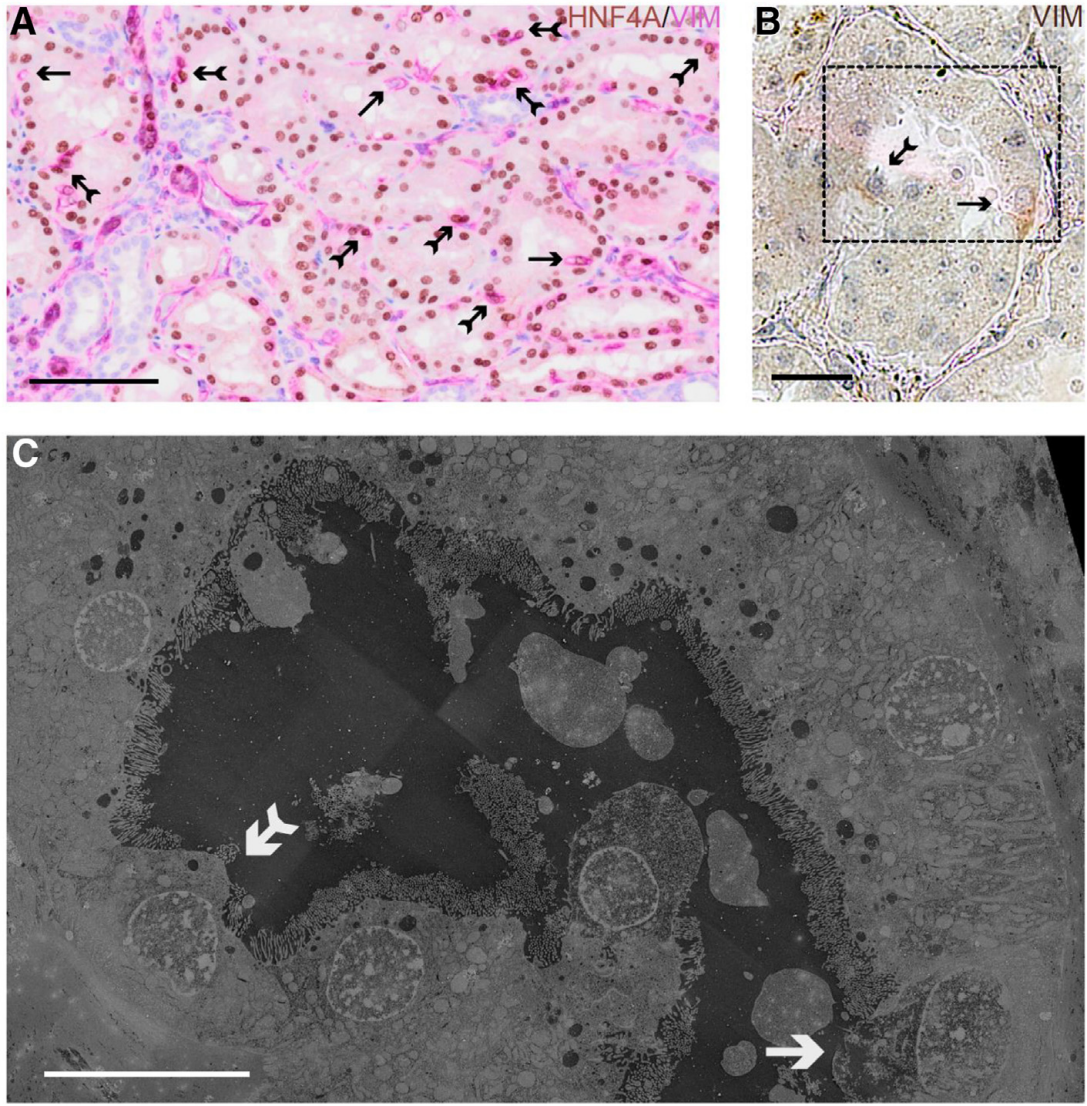
Scattered tubular cells in normal human kidney show reduced hepatocyte nuclear factor 4α (HNF4A) expression. **A:** Immunohistochemical double staining of human kidney tissue for HNF4A (brown) and vimentin (VIM; pink) shows double positivity for most VIM-positive proximal tubular cells (**fletched arrows**). A subpopulation of the VIM-positive cells displays loss of HNF4A expression (**arrows**). **B** and **C:** Representative images from correlative light and electron microscopy of human kidney tissue reveal that most VIM-positive cells display a reduced brush border and decreased mitochondrial content (**fletched arrow**), whereas a few VIM-positive cells show complete absence of brush border and mitochondria (**arrow**). Scale bars: 100 μm (**A**); 50 μm (**B**); 25 μm (**C**).

To further explore the possibility that HNF4A loss may be a stereotypic response of injured human PT cells, the next set of experiments focused on STCs, a cell type that resides among PT cells in healthy human kidneys and stains positive for VIM and other injury markers.^4^^,^^6^^,^^14^^,^^15^ This cell population expands in disease, because either STCs represent progenitor cells that proliferate^5^^,^^10^ or dedifferentiation of PT cells involves gain of phenotypic properties typical of STCs.^6^^,^^8^^,^^9^ Immunohistochemical double staining of healthy human kidney for HNF4A and VIM revealed that most VIM-positive cells co-expressed HNF4A (Figure 2A) (approximately 4% of PT cells). However, a smaller number of VIM-positive cells with weaker or complete loss of HNF4A expression were also observed (Figure 2A) (approximately 1% of PT cells). To investigate this further, a method for correlative light and electron microscopy (CLEM) was developed where VIM-positive cells could be localized by light microscopy using immunohistochemistry and their ultrastructural features studied using scanning electron microscopy. CLEM demonstrated that the VIM-positive cells displayed a gradual loss of brush border and mitochondria (Figure 2, B and C). Only a minority of the VIM-positive cells exhibited complete loss of brush border and mitochondria (Figure 2, B and C). It was hypothesized that the HNF4A^+^/VIM^+^ cells represent the cells with partial loss of brush border, whereas the HNF4A^−^/VIM^+^ cells represent cells with complete loss of brush border. Furthermore, it was hypothesized that loss of the PT phenotype was correlated with development of the STC phenotype and that the HNF4A^+^/VIM^+^ cells represented an intermediate form between these two phenotypes.

### Mitochondrial Load Decreases and the Tight Junction Protein CLDN1 Is Up-Regulated during Acute and Chronic Kidney Injury

**Figure 3:**
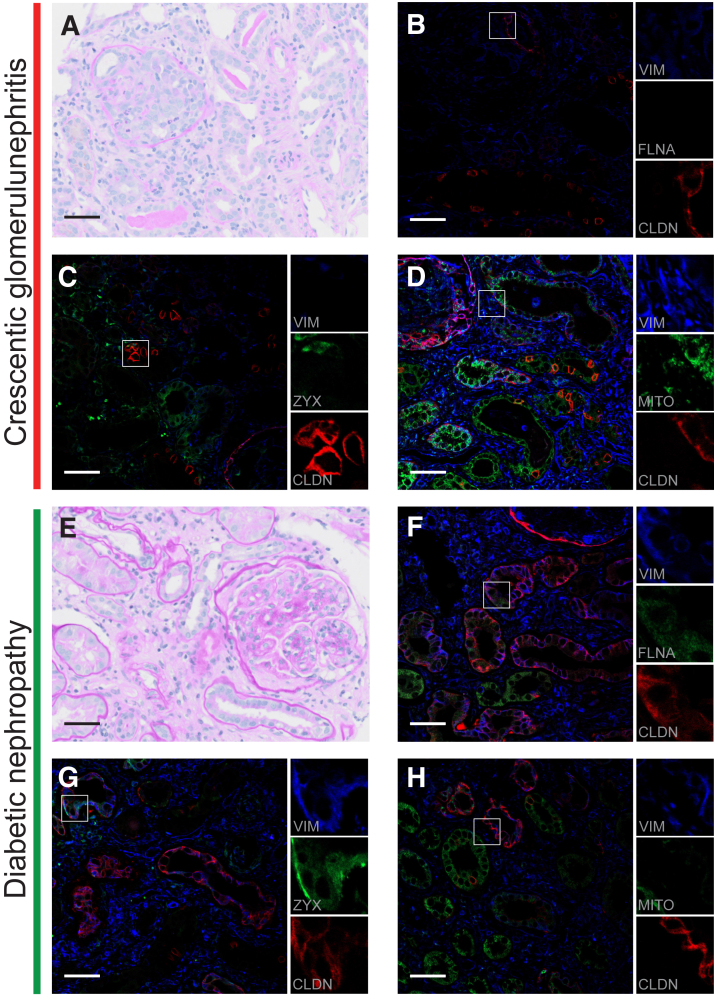
Vimentin (VIM)–positive tubular cells express tight junction proteins, F-actin–associated proteins, and reduced mitochondrial load. **A:** Hematoxylin/eosin staining of human biopsy material from a case of crescentic glomerulonephritis. Tubular injury is visible as reduction of tubular epithelial cellular height and sloughing of apical plasma membranes. **B:** The tight junction protein claudin 1 (CLDN1) colocalizes with VIM in injured proximal tubular (PT) cells. Filamin A (FLNA) displays weak positivity. **C:** Zyxin (ZYX) and CLDN1 colocalize to vimentin-positive cells. **D:** VIM and CLDN1 are expressed in moderately injured tubules in parallel with a decrease in mitochondrial number (MITO). In more severely injured tubular segments, an even more pronounced reduction is seen. **E:** A case of severe diabetic nephropathy with reduced number of PTs and interstitial fibrosis. The glomerulus is undergoing sclerosis. **F:** Injured tubules coexpress CLDN1, FLNA, and VIM. **G:** Coexpression of VIM, ZYX, and CLDN1 in injured tubules. **H:** Reduction of MITOs in cells expressing VIM and CLDN1. Scale bar = 50 μm (**A**–**H**).

To further study the phenotypic changes associated with gain of vimentin and loss of HNF4A in kidneys affected by crescentic glomerulonephritis and diabetic nephropathy (Figure 3, A and E), colocalization of VIM with a general mitochondrial marker was performed. Also, vimentin was colocalized to the tight junction CLDN1, the filamentous actin-associated protein FLNA, and finally the focal adhesion plaque-associated protein ZYX. Increased VIM expression was strongly associated with a parallel decrease of mitochondrial load (Figure 3, D and H). Increased expression of VIM was associated with *de novo* expression of the tight junction protein CLDN1 (Figure 3, B and F). Furthermore, tubular injury was associated with expression of FLNA and ZYX that colocalized to VIM in injured tubular segments (Figure 3, C and G).

### HNF4A Is Down-Regulated in Cultured Primary Proximal Tubular Epithelial Cells

**Figure 4:**
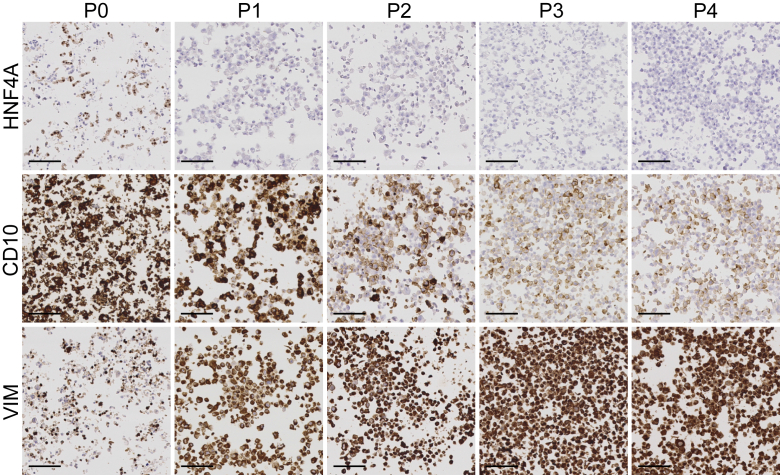
Primary proximal tubular epithelial cells (PTECs) lose expression of proximal tubular markers during culture. PTECs harvested directly following tissue dissociation, preseeding (P0), and at consecutive passages (P1 to P4), followed by immunohistochemical staining, show a rapid reduction of hepatocyte nuclear factor 4α (HNF4A) expression. Expression of the proximal tubular marker CD10 is also down-regulated, but this is more protracted in time. In contrast, the scattered tubular cell marker vimentin (VIM) is rapidly and highly induced in cultured PTECs. *n* = 3. Scale bars = 100 μm.

Primary PTECs adopt an STC-like phenotype during culture, characterized by an increased expression of SOX9, VIM, and other markers related to injury.^15^ To test whether this transition involves loss of HNF4A, cells were harvested immediately after tissue dissociation (P0) and at consecutive passages (P1 to P4), and immunohistochemistry was performed for the PT markers HNF4A and CD10, and the STC marker VIM (Figure 4). A rapid reduction of HNF4A expression was evident already at P0, with a considerably lower percentage of positive cells than in tissue (Figure 2A). In the following passages, the cells lost HNF4A positivity altogether (Figure 3). The expression of the structural PT marker CD10 was to a large degree maintained at P0, confirming that the cultured cells were of proximal tubular origin. With increasing passage number, CD10 was down-regulated. Conversely, the VIM expression was broadly induced already at low passages and accentuated over time in the cultured cells. Purchased renal PTECs from Lonza exhibited a staining pattern that matched serially passaged PTECs, and a negligible fraction of renal PTECs displayed HNF4A positivity (Supplemental Figure S1). Collectively, these data show that primary PTECs rapidly lose essential PT features during culture, such as expression of HNF4A and CD10. Simultaneously, the STC phenotype is induced, as seen by increased expression of VIM. Thus, loss of HNF4A is a stereotypic response of PTECs to various challenges *in vivo* and *in vitro*.

### HNF4A Transduction of Primary Cells Restores the Proximal Tubular Phenotype

**Figure 5:**
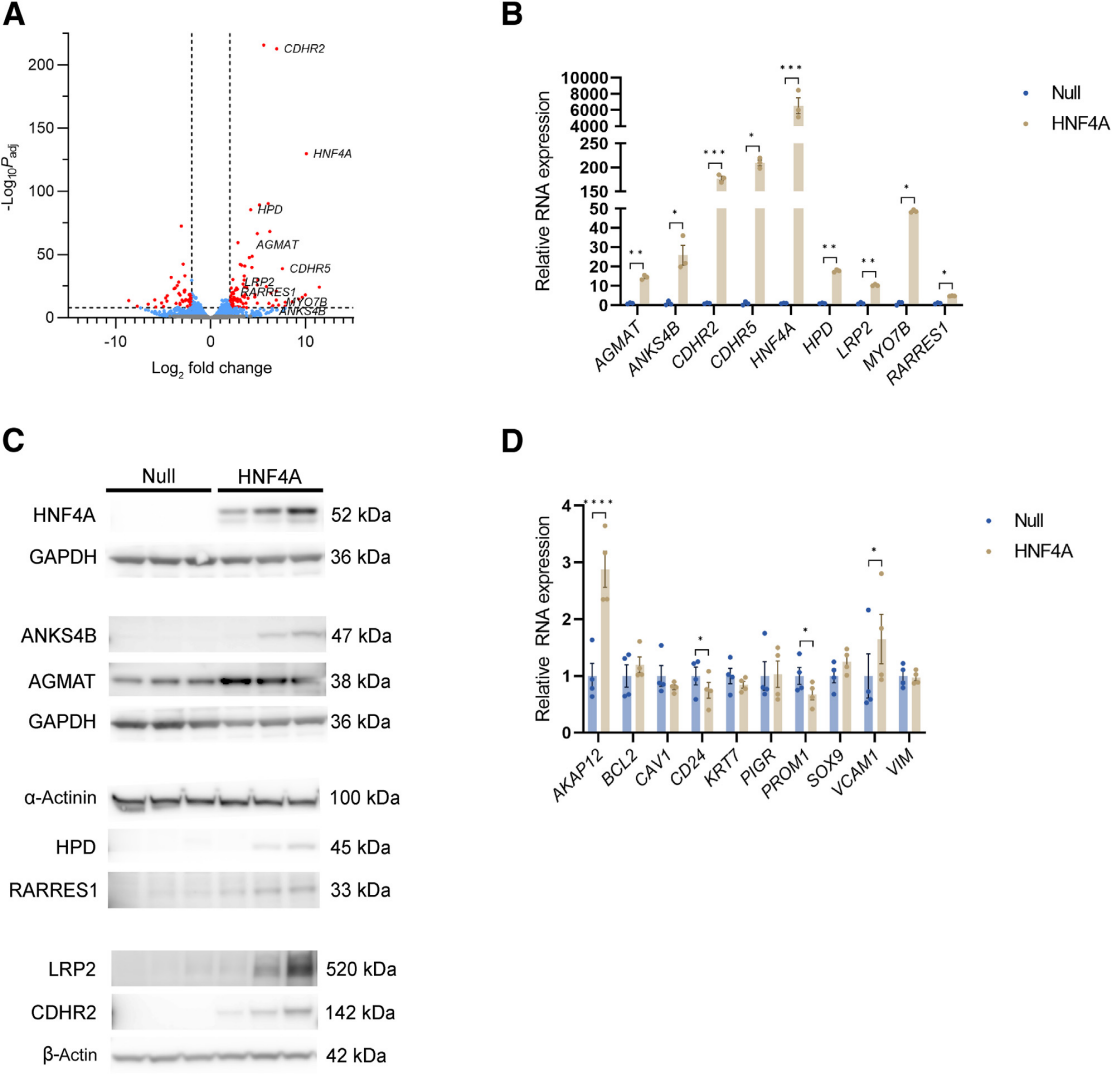
Differentially expressed genes (DEGs) after hepatocyte nuclear factor 4α (HNF4A) transduction of primary proximal tubular epithelial cells are associated with the proximal tubular phenotype. **A:** Volcano plot of RNA-sequencing (RNA-seq) data reveals 2033 significant DEGs in HNF4A-transduced cells compared with Null (blue and red). After further filtering, 130 genes remained [adjusted *P* (*P*_adj_) < 10^−8^, |log_2_ fold change| > 2; marked by **dashed black line** and gene transcript positions in red]. Of these, eight genes (*AGMAT*, *ANKS4B*, *CDHR2*, *CDHR5*, *HPD*, *LRP2*, *MYO7B*, and *RARRES1*) were selected for further confirmation based on their role as known HNF4A target genes, function, and histologic expression pattern. **B:** Quantitative PCR of HNF4A and the selected genes demonstrates significant up-regulation at the RNA level in all cases 4 days after HNF4A transduction. Statistical significance is determined by paired *t*-test. **C:** Western blot analysis shows up-regulation at the protein level of HNF4A, ankyrin repeat and sterile α motif domain-containing 4B (ANKS4B), agmatinase (AGMAT), 4-hydroxyphenylpyruvate dioxygenase (HPD), retinoic acid receptor responder 1 (RARRES1), low-density lipoprotein receptor-related protein 2 (LRP2), and cadherin-related family member 2 (CDHR2) 8 days after HNF4A transduction. **D:** RNA-seq data analysis displays modest alterations and no systematic pattern of expression of established scattered tubular cell markers [A-kinase anchoring protein 12 (*AKAP12*), B-cell lymphoma 2 (*BCL2*), caveolin 1 (*CAV1*), *CD24*, keratin 7 (*KRT7*), polymeric Ig receptor (*PIGR*), prominin 1 (*PROM1*), SRY box transcription factor 9 (*SOX9*), vascular cell adhesion molecule 1 (*VCAM1*), and vimentin (*VIM*)] following HNF4A transduction. Data are presented as means ± SEM (**B** and **D**). *n* = 3 (**B**); *n* = 4 (**D**). ∗*P* < 0.05, ∗∗*P* < 0.01, and ∗∗∗*P* < 0.001 (**B**); ∗*P*_adj_ < 0.05, ∗∗∗∗*P*_adj_ < 0.0001 (**D**). *CDHR5*, cadherin-related family member 5; GAPDH, glyceraldehyde-3-phosphate dehydrogenase; *MYO7B*, myosin VIIB.

Attempting to restore the lost PT phenotype in cultured primary PTECs, cells were transduced with HNF4A adenovirus. Null adenovirus was used as control and RNA was collected 4 days after transduction. RNA-sequencing (RNA-seq) analysis resulted in 2033 significant DEGs (Figure 4 and Supplemental Table S1). The list of DEGs was further filtered (adjusted *P* < 10^−8^, |log_2_ fold change| > 2), resulting in 130 remaining genes (Figure 5A). On the basis of their known roles as HNF4A target genes, function, and histologic expression in the Human Protein Atlas (*https://www.proteinatlas.org*,^36^ last accessed May 28, 2024), eight genes (*AGMAT*, *ANKS4B*, *CDHR2*, *CDHR5*, *HPD*, *LRP2*, *MYO7B*, and *RARRES1*) were selected for confirmation. Quantitative PCR confirmed that the RNA expression of all eight genes was significantly up-regulated after HNF4A overexpression (Figure 5B). Next, the protein expression of the selected genes was analyzed by Western blot analysis. Six of the selected genes (*AGMAT*, *ANKS4B*, *CDHR2*, *HPD*, *LRP2*, and *RARRES1*) also showed up-regulation at protein level after HNF4A overexpression (Figure 5C). Notably, most of these proteins were not detectable in the null samples, although they are typically expressed by PT cells *in situ*, underlining dedifferentiation of PT cells in culture. No previously identified STC markers were found in the filtered list (adjusted *P* < 10^−8^, |log_2_ fold change| > 2) of suppressed genes following HNF4A transduction. Therefore, the fate of 10 histologically validated STC markers (*AKAP12*, *BCL2*, *CAV1*, *CD24*, *KRT7*, *PIGR*, *PROM1*, *SOX9*, *VCAM1*, and *VIM*) was examined in the RNA-seq data (Figure 5D). Some of them were modestly reduced, whereas some were modestly increased (at adjusted *P* < 0.05). Hence, the STC phenotype, characterized by expression of essentially all these markers, does not appear to be suppressed by HNF4A overexpression, even if HNF4A overexpression may contribute to down-regulation of some STC markers (*CD24* and *PROM1*).

**Figure 6:**
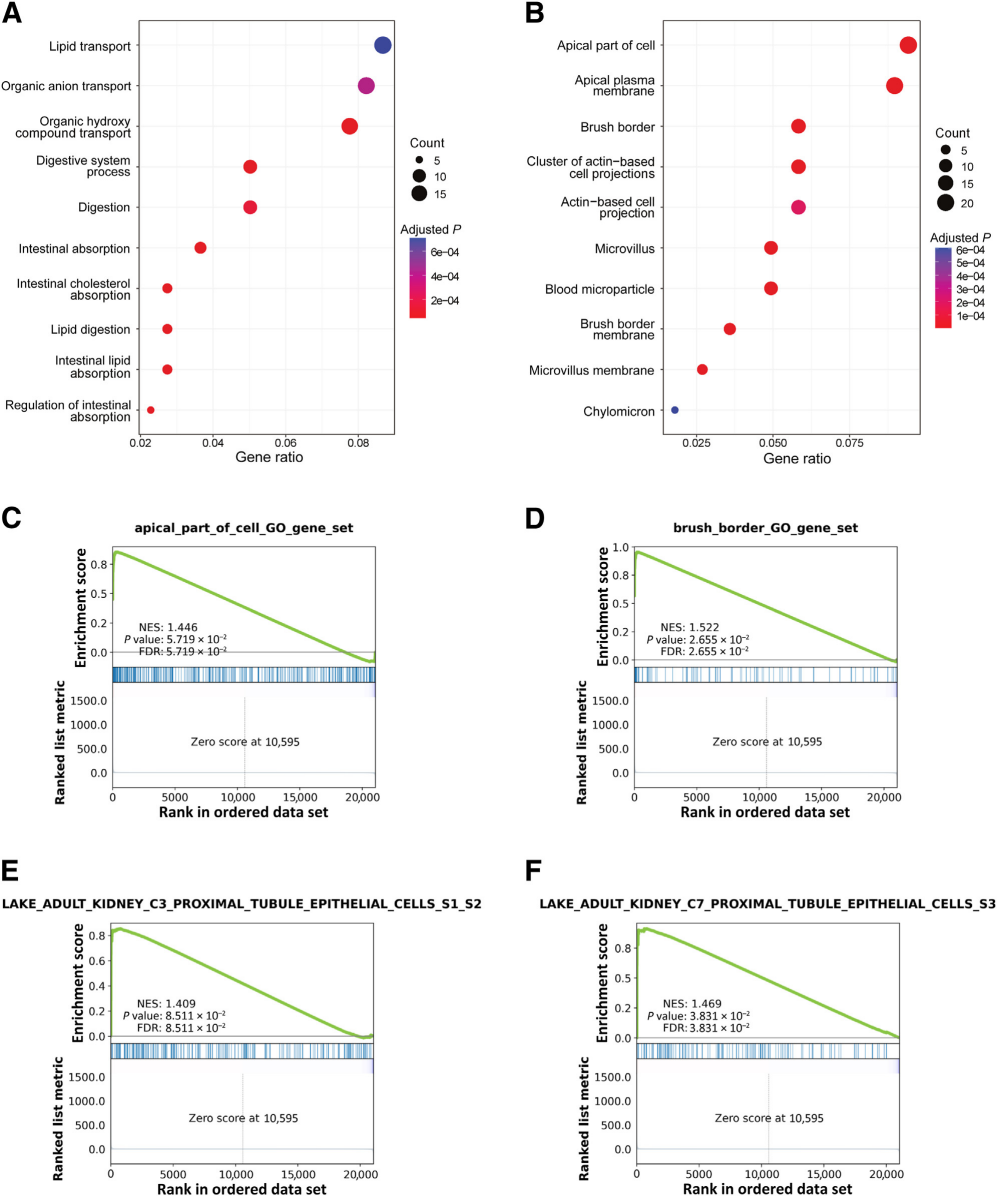
The proximal tubular phenotype is partially restored in primary proximal tubular epithelial cells (PTECs) after hepatocyte nuclear factor 4α (HNF4A) transduction. **A:** Gene Set Enrichment Analysis (GSEA) of differentially expressed genes (DEGs) following HNF4A transduction of PTECs using Gene Ontology biological process displays overrepresentation of gene sets associated with transport and absorption. **B:** GSEA using Gene Ontology cellular component (GO-CC) shows overrepresentation of gene sets linked to brush border. **C** and **D:** Enrichment plots of GO-CC gene sets apical part of cell and brush border, respectively, demonstrate significant enrichment. **E** and **F:** Gene sets of proximal tubule S1 to S2 and S3 segments, respectively, from single-nucleus RNA sequencing of adult human kidney^34^ were shown to be significantly enriched among DEGs in HNF4A-transduced cells (figures from our data analysis). False discovery rate (FDR) < 0.25 is considered statistically significant. NES, normalized enrichment score.

GSEA of DEGs was performed using GO terms within biological process and cellular component. Overrepresented gene sets from GO biological process included gene sets within transport and absorption (Figure 6A and Supplemental Table S2), which are typical features of the PT cells *in situ*. GSEA using GOrilla also revealed that the GO biological process gene sets regulation of microvillus length (*P* = 2.41 × 10^−6^, FDR = 1.02 × 10^−3^) and brush border assembly (*P* = 4.40 × 10^−6^, FDR = 1.48 × 10^−3^) were significantly enriched and up-regulated in the HNF4A-transduced cells (Supplemental Table S3). GSEA of GO cellular component resulted in overrepresentation of gene sets that were directly or indirectly connected to the brush border, such as apical part of cell, apical plasma membrane, brush border, cluster of actin-based projections, actin-based cell projection, microvillus, brush border membrane, and microvillus membrane (Figure 6B and Supplemental Table S4). GSEA using GOrilla mirrored these findings (Supplemental Table S5). Enrichment plots of the gene sets apical part of cell and brush border further strengthened the results (Figure 6, C and D). Notably, seven of the evaluated DEGs were found among the enriched gene sets. *ANKS4B*, *CDHR2*, *CDHR5*, and *MYO7B* were generally included among the gene sets linked to the brush border (Supplemental Table S4). *LRP2* was also included in some gene sets related to the brush border but mainly in gene sets associated with transport, whereas *AGMAT* and *HPD* were included in the gene sets α-amino acid metabolic process (*P* = 1.97 × 10^−5^, FDR = 3.01 × 10^−3^) and cellular amino acid metabolic process (*P* = 6.65 × 10^−5^, FDR = 6.00 × 10^−3^) (Supplemental Table S2). Furthermore, gene sets of the PT S1 to S2 and S3 segments from single-nucleus RNA-seq of adult human kidney^34^ were significantly enriched among the DEGs in the HNF4A-transduced cells (Figure 6, E and F). Taken together, these data demonstrate that HNF4A overexpression in cultured primary PTECs restores essential aspects of the proximal tubular phenotype, including functions related to transport, absorption, and formation of brush border.

## Discussion

This study demonstrated that partial, or more often, complete depletion of the transcription factor HNF4A characterizes a range of acute and chronic kidney diseases. Moreover, a subset of the STC population found in healthy human kidney exhibited loss of HNF4A. Challenge of PT cells by isolation and subculture caused rapid and robust HNF4A depletion. Although the basis for HNF4A loss in these conditions remains unknown, it is evidently a stereotypic response that faithfully recapitulated the essential features of PT injury.

Immunohistochemical double staining for HNF4A and VIM of human kidney biopsies from pathophysiologically and temporally diverse kidney diseases, such as crescentic nephritis and diabetic nephropathy, displayed extensive increases in VIM expression combined with loss of HNF4A expression. During chronic kidney disease, stretches of cells with loss of HNF4A could be observed. Acute and severe kidney injury resulted in similar tubular responses, with the important observation that sloughing of HNF4A^−^/VIM^+^ PT cells into the PT lumen was mainly associated with crescentic nephritis.

In healthy human kidney, staining for HNF4A and VIM revealed a double-positive cell population, indicative of an intermediate phenotype between the HNF4A-positive PT phenotype and the VIM-positive STC phenotype. Results from CLEM strengthened this observation, by demonstrating a VIM-positive cell population with diminished, but not completely absent, brush border as well as reduced, but not lost, mitochondrial content. The CLEM method is complex and based on scanning electron microscopy, which results in a less distinct morphology than transmission electron microscopy. Therefore, tissue sections were stained for mitochondria and VIM. A graded decrease of mitochondrial load was associated with the VIM-positive STC phenotype. Vimentin is a marker for epithelial-mesenchymal transition, often in conjunction with loss of E-cadherin expression. Proximal tubules do not express E-cadherin, precluding detection of loss. Staining for CLDN1 was performed instead. This tight junction protein is expressed by parietal epithelial cells of the Bowman capsule. Similarities in marker distribution between STCs and PECs have previously been observed. A *de novo* expression of CLDN1 in the injured PT cells was observed. FLNA and ZYX, proteins associated with the filamentous actin cytoskeleton, are part of the STC phenotype. Up-regulation of tight junction proteins and cytoskeletal rearrangement in filamentous direction suggest that the STC phenotype is not an example of epithelial-mesenchymal transition in the classic sense. Fixed primary PTECs, harvested at different passages of *in vitro* propagation and subjected to immunostaining, were used to follow the process of dedifferentiation during culture, with loss of PT markers and up-regulation of STC markers with increasing passage number. HNF4A loss in this setting is in line with recent work that demonstrates that the phenotype of cultured primary PTECs is closer to failed repair rather than healthy PT.^37^ The histologic findings regarding intermediate phenotypes between PT and STC are strengthened by observations from single-cell RNA-seq data from adult human kidney, which identify subclusters with varying expression of STC markers and mature PT markers.^16^^,^^17^ These subclusters could either reflect PT cells undergoing dedifferentiation or STCs undergoing redifferentiation. Nevertheless, these findings suggest that the STCs represent a dedifferentiated phenotype, rather than a progenitor population. In addition, Muto et al^16^ observe high activity of HNF4A in the PT cluster, whereas it was reduced in the VCAM1-positive cluster, suggesting a role for HNF4A in the transition between the two phenotypes, consistent with results of the present work. Studies on enhancer regulation also find activation of HNF4A during kidney repair, but because HNF4A is expressed in liver and pancreas, Wilflingseder et al suggest that a panel of transcription factors is necessary for kidney-specific regulation, including HNF4A and the glucocorticoid receptor.^38^ A general down-regulation of STC markers in HNF4A-transduced cells from the filtered list of DEGs was not observed (adjusted *P* < 10^−8^, |log_2_ fold change| > 2). The examination of 10 previously established STC markers in our RNA-seq data did not show any consistency in induction or suppression following HNF4A transduction, suggesting that other transcription factors might be required for driving the STC phenotype and possibly the repair process. The transcription factor nuclear factor of activated T-cells 5 (NFAT5) was recently identified as a driver of the failed repair or maladaptive PT state based on regularized regression analysis of single-cell multiomic sequencing data.^37^ Ledru et al^37^ also found down-regulation of genes associated with failed repair and fibrosis following NFAT5 knockdown, suggesting that this could possibly be combined with HNF4A overexpression to drive successful repair.

Efforts have been made in improving *in vitro* models of PTECs, because they typically lose the expression of many key genes and features of proximal tubules *in situ* during traditional two-dimensional culture.^23^, ^24^, ^25^ Culture of PTECs on Transwell inserts preserves the expression of renal transporters,^39^^,^^40^ whereas kidney microphysiological systems show lower expression of kidney injury molecule-1, indicating a less injured or stressed state, compared with static two-dimensional culture.^41^^,^^42^ The application of fluid shear stress in microphysiological systems mimics the physiological conditions that PTECs are exposed to *in vivo* and helps maintain the PT phenotype. The morphology of the cells in microphysiological systems assumes the normal columnar shape and height of epithelial cells.^43^ Furthermore, cells exposed to fluid shear stress can form primary cilia, which is important for mechanosensing and regulation of tubular morphology.^41^^,^^43^ In this study, a simple *in vitro* model was presented where PTECs are cultured in a traditional two-dimensional setup, and where HNF4A overexpression via adenoviral transduction imparts key features of the PT phenotype seen *in vivo*.

Studies in murine epithelial tissues show that *Hnf4* drives brush border formation across multiple tissues, such as kidney, intestine, and yolk sac.^21^ Using CLEM, sloughing of brush border membranes in VIM-positive was observed and thus probably HNF4A-negative cells. Furthermore, the results from GSEA of DEGs in HNF4A-transduced cells were in line with this. Enrichment of gene sets associated with brush border, microvillus, and apical part of cell, as well as regulation of microvillus length and brush border assembly, was found. Genes involved in these gene sets, *ANKS4B*, *CDHR2*, *CDHR5*, and *MYO7B*, all part of the intermicrovillar adhesion complex (IMAC), were up-regulated at the RNA level following HNF4A transduction. Together with *USH1C* and *CALML4*, they are important for brush border assembly and organization in the intestinal epithelium, localizing in the distal tips of microvilli.^44^, ^45^, ^46^, ^47^, ^48^ More recently, myosin VB (MYO5B) was presented as a transporter of the IMAC components, and loss of MYO5B resulted in shortened microvilli and disorganized brush border.^49^ Notably, the expression of *USH1C*, *CALML4*, and *MYO5B* was significantly up-regulated in HNF4A-transduced cells. Up-regulation of all the IMAC components following HNF4A overexpression in PTECs suggests that HNF4A drives the formation of the IMAC and that these genes are involved in brush border assembly in kidney epithelium. A recent study presented another IMAC located at the proximal base region of microvilli in intestinal epithelium, composed of transmembrane and immunoglobulin domain-containing 1 (TMIGD1), SLC9A3 regulator 1 (SLC9A3R1; alias EBP50), and SLC9A3 regulator 2 (SLC9A3R2; alias E3KARP).^50^ None of these components was found among the DEGs in the current RNA-seq data, indicating that this second IMAC is not driven by HNF4A or that other components are involved in kidney epithelium.

HNF4A has an established role in controlling reabsorption, transport, and metabolism in PTECs.^20^ RNA-seq of HNF4A-transduced cells confirmed this by showing enrichment of gene sets related to transport, where *LRP2*, encoding an endocytic receptor for filtered proteins, alias megalin, was included. Up-regulation of *LRP2* at both the RNA and protein level after HNF4A transduction was validated in agreement with previous reports regarding *LRP2* as a target gene of HNF4A.^51^ Recent studies present LRP2 as a master regulator of ion transport, metabolism, and endocytosis in the PT, and show the involvement of cubilin (CUBN) and DAB adaptor protein 2 (DAB2) as additional components in this endocytic pathway.^52^ These components drive the endocytic flux but are not essential for the physical integrity of the endocytic pathway.^53^ However, *CUBN* and *DAB2* were not found among the DEGs following HNF4A transduction in our data. Interestingly, *CUBN* was presented as a target gene of HNF4A in a recent publication on HNF4A-knockout kidney organoids,^54^ where Yoshimura et al^54^ showed direct binding of HNF4A to the *CUBN* promoter and enhancer regions using CUT&RUN sequencing and further confirmed direct regulation via clustered regularly interspaced short palindromic repeats (CRISPR)–mediated transcription activation. HNF4A probably drives expression of components involved in endocytosis as part of its regulation of the PT phenotype, but different model systems might be required to highlight various components. RNA-seq and GSEA data of HNF4A-knockout kidney organoids from Yoshimura et al^54^ are in line with our data from HNF4A-transduced PTECs, displaying terms within lipid metabolism, transport, and brush border.

The current findings highlight the process of dedifferentiation of PTECs, associating with loss of HNF4A, and development to the STC phenotype, further strengthening the hypothesis that the STCs represent an induced phenotype. Moreover, major features of the PT phenotype could be restored in two-dimensional PTEC culture via HNF4A re-expression, including expression of genes involved in metabolism, transport, and brush border formation. Future improvements of this model may include the combination of HNF4A transduction with culture on Transwell inserts, application of fluid shear stress, stimulation with glucocorticoids, and NFAT5 knockdown, which could further improve morphologic and functional aspects of the PTECs. Interestingly, induction of components of the IMAC, responsible for brush border assembly and organization in intestinal epithelium, were identified following HNF4A overexpression. Further studies are needed to confirm that these factors are driven by HNF4A and that the IMAC has the same role in kidney epithelium.

## Disclosure Statement

None declared.

## Supplemental Data

Supplemental Table S5

Supplemental Figure S1 Commercial primary proximal tubular epithelial cells (PTECs) lack expression of proximal tubular markers. Renal PTECs (RPTECs), purchased from Lonza, harvested at passage 2 to 5 (P2 to P5), and subjected to immunohistochemical staining, show an expression pattern consistent with that in the current cultured primary PTECs [ie, reduced expression of hepatocyte nuclear factor 4α (HNF4A) and CD10, and high vimentin (VIM) expression]. Some HNF4A-positive cells could be observed among the Lonza RPTECs. Scale bars = 100 μm.
