# Supplementary figures and images for: Injured Proximal Tubular Epithelial Cells Lose Hepatocyte Nuclear Factor 4α Expression Crucial for Brush Border Formation and Transport
Source: Am J Pathol. 2025 Feb 13;195(5):845–60. doi: 10.1016/j.ajpath.2025.01.011 (PMC12179525; doi:10.1016/j.ajpath.2025.01.011)

Supplemental Figure S1

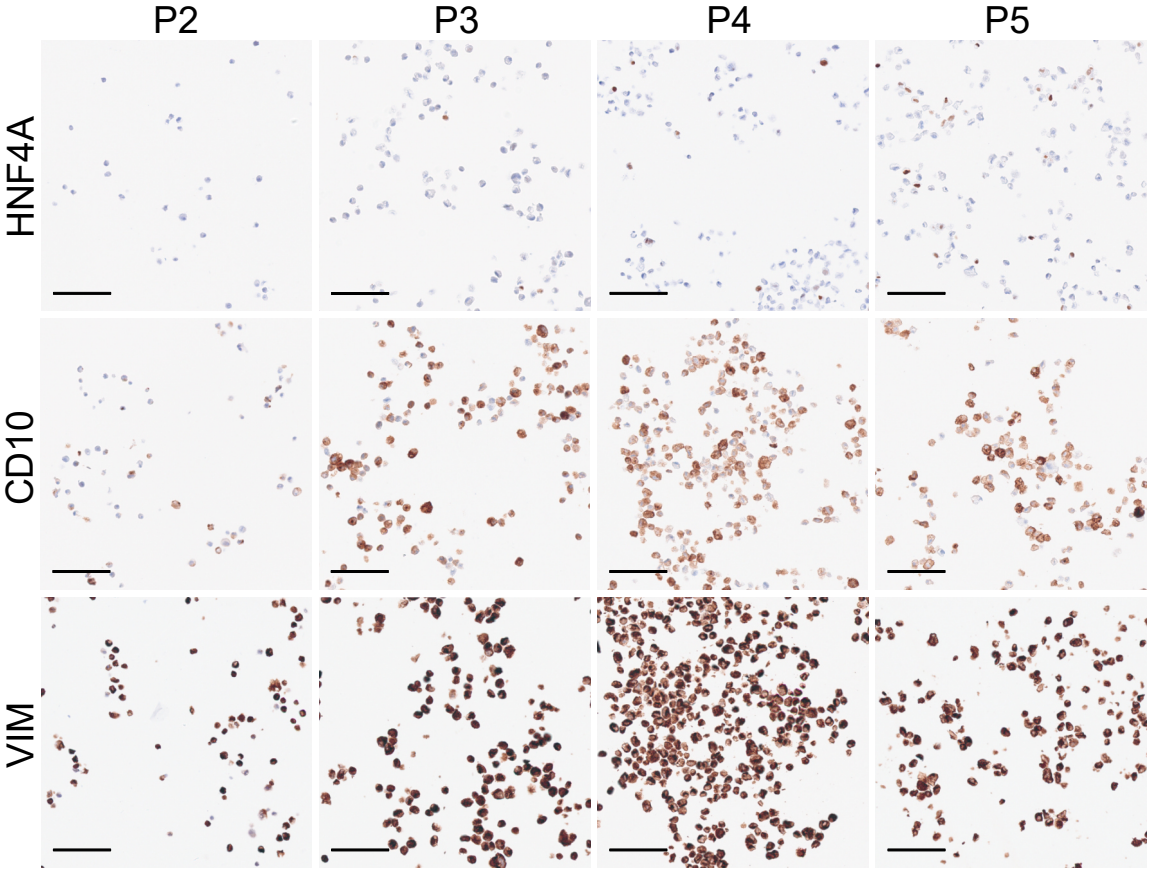

Supplement: Supplemental Figure S1 — Commercial primary proximal tubular epithelial cells (PTECs) lack expression of proximal tubular markers. Renal PTECs (RPTECs), purchased from Lonza, harvested at passage 2 to 5 (P2 to P5), and subjected to immunohistochemical staining, show an expression pattern consistent with that in the current cultured primary PTECs [ie, reduced expression of hepatocyte nuclear factor 4α (HNF4A) and CD10, and high vimentin (VIM) expression]. Some HNF4A-positive cells could be observed among the Lonza RPTECs. Scale bars = 100 μm. [file mmc6.pdf]
